# Exploiting CRISPR/Cas: Interference Mechanisms and Applications

**DOI:** 10.3390/ijms140714518

**Published:** 2013-07-12

**Authors:** Hagen Richter, Lennart Randau, André Plagens

**Affiliations:** Prokaryotic Small RNA Biology, Max Planck Institute for Terrestrial Microbiology, Karl-von-Frisch-Straße 10, 35043 Marburg, Germany; E-Mails: hagen.richter@mpi-marburg.mpg.de (H.R.); lennart.randau@mpi-marburg.mpg.de (L.R.)

**Keywords:** CRISPR, crRNA, Cas9, Cascade, interference, genome editing, RGEN, TALEN, ZNF

## Abstract

The discovery of biological concepts can often provide a framework for the development of novel molecular tools, which can help us to further understand and manipulate life. One recent example is the elucidation of the prokaryotic adaptive immune system, clustered regularly interspaced short palindromic repeats (CRISPR)/CRISPR-associated (Cas) that protects bacteria and archaea against viruses or conjugative plasmids. The immunity is based on small RNA molecules that are incorporated into versatile multi-domain proteins or protein complexes and specifically target viral nucleic acids via base complementarity. CRISPR/Cas interference machines are utilized to develop novel genome editing tools for different organisms. Here, we will review the latest progress in the elucidation and application of prokaryotic CRISPR/Cas systems and discuss possible future approaches to exploit the potential of these interference machineries.

## 1. Introduction

The clustered regularly interspaced short palindromic repeats (CRISPR)/CRISPR-associated (Cas) systems are found in many bacteria and nearly all archaea and constitute an adaptive immune system that recognizes and prevents viral attacks. A breakthrough for the basic understanding of the immunity mechanism was achieved by Barrangou and coworkers, who could show that *Streptococcus thermophilus* can acquire resistance against a bacteriophage by integrating a genome fragment of an infectious virus into its CRISPR locus [1]. A CRISPR cluster is a genomic DNA element that consists of a series of short repeat sequences (typically 24–37 bp) that are separated by unique spacer sequences of similar length [2]. These sequences are often fragments derived from a viral genome, illustrating the genetic memory of previous infections [3,4]. The second part of the CRISPR/Cas machinery is encoded in the Cas genes, and the Cas proteins fulfill essential functions within the immunity mechanism.

**Figure 1:**
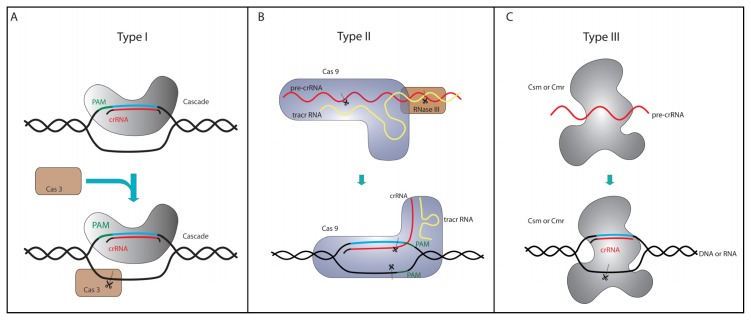
The interference step in the three clustered regularly interspaced short palindromic repeats (CRISPR)/CRISPR-associated (Cas) types. Indicated for all three systems are the targeted DNA region (blue), the targeting crRNA (red) and the protospacer adjacent motif (PAM, green). In type I systems (**A**), the invading DNA is recognized by the Cascade:crRNA complex. The PAM motif promotes the identification of the foreign DNA. Subsequently, the nuclease, Cas3, is recruited and degrades the target DNA. Type II systems (**B**) require only Cas9 for interference and do not rely on a multi-protein complex. Complex formation of Cas9, tracrRNA and pre-crRNA enables RNase III to mature crRNAs. The resulting complex of Cas9 and the tracrRNA:crRNA duplex recognizes the invading nucleic acid, and the Cas9 nuclease generates blunt-ended cleavage of both DNA strands. In type III systems (**C**), a multi-protein complex (Csm or Cmr) or Cas6 processes pre-crRNA into mature crRNA. The complex-bound crRNA recognizes invading DNA (Csm) or RNA (Cmr), resulting in target degradation.

The CRISPR/Cas defense response is classified into three main stages, which are conserved throughout all CRISPR systems. In the first stage, termed adaptation, the injected viral DNA is recognized, and a fragment of this DNA inserted as a new spacer into a host CRISPR array. A short conserved sequence (2 to 5 nt), called the protospacer adjacent motif (PAM), flanks the spacer sequence in the viral genome (termed protospacer) and, thus, determines the targets of most CRISPR/Cas systems [5,6]. A new spacer is always integrated at the AT-rich leader site of a CRISPR, which is proposed to contain specific sequence elements that direct spacer DNA addition [7,8]. The detailed mechanism of spacer acquisition is only partially understood, but the highly conserved proteins, Cas1 and Cas2, were identified as key players in this process [9,10]. The second stage is characterized by the transcription of a CRISPR cluster into a long precursor-crRNA (pre-crRNA). Transcription is controlled by sequences in the leader region that contains promoter elements and possible binding sites for regulatory proteins [11,12]. In many systems, the long pre-crRNA transcript is processed by a Cas endonuclease (Cas6) into short crRNAs [13–17]. A typical crRNA generated by Cas6 cleavage contains the complete spacer sequence flanked by an 8 nt 5′-hydroxyl repeat tag and a 2′–3′ cyclic phosphate repeat end [17,18]. In some cases, 3′ termini of mature crRNAs are trimmed further, but the mechanism is unknown. In the final stage, the interference reaction, these mature crRNAs are incorporated into a larger Cas protein complex and used to target and degrade the viral DNA during a repeated attack (Figure 1) [19–21]. In the next section, we will focus on the molecular details of different interference mechanisms with diverse sets of Cas proteins and multi-subunit complexes. These crRNA/Cas protein complexes exhibit a large potential for the development of genetic tools.

## 2. Three Strategies to Cope with Viruses

CRISPR/Cas systems are identified in diverse bacterial and archaeal species, often living in the most extreme ecological niches. Some basic principles of the CRISPR/Cas systems are conserved within all prokaryotes, but the plethora of identified Cas protein families reflects the divergence of mechanistic details during evolution. The continuous co-evolution of viruses and their hosts led to the emergence of anti-CRISPR measures in viruses [22], which might explain the necessity for CRISPR/Cas diversification. Computational studies classified all CRISPR/Cas systems into three types and at least ten subtypes [23–25].

Type I CRISPR/Cas systems are found in both bacteria and archaea and are comprised of six different subtypes (subtypes I-A to I-F). The essential and significantly conserved marker protein in the interference reaction is Cas3, which contains a HD phosphohydrolase domain and a DExH-like helicase domain [24,25]. Both domains are also found to be encoded separately by two discrete genes. These two domains have been shown to unwind dsDNA (helicase domain) and cleave ssDNA (HD nuclease domain), depending on ATP and Mg^2+^ ions [26–28]. Cas3 interacts with a complex of different Cas proteins that bind and deliver the crRNA (Figure 1A). This complex is termed Cascade (CRISPR-associated complex for antiviral defense) and is best studied for the type I-E system from *Escherichia coli* [18,19]. The type I-E Cascade was shown to bind the short crRNAs, which are utilized to recognize DNA target sequences that are complementary to the spacer sequence within the crRNA. Subsequently, Cascade recruits Cas3 to degrade the targeted viral DNA molecule, relying on negatively supercoiled DNA [29,30]. The first 6–12 nt of the crRNA spacer are most important for target binding and are termed the seed sequence [20,21]. The point mutation of single seed sequence nucleotides results in drastic binding defects. However, mismatches in the crRNA spacer sequence following the seed sequence are tolerated, and the mutated crRNA can still be bound [31]. To avoid that the Cascade:Cas3 complex degrades the host genomic encoded CRISPR cluster, it has to be ensured that the 5′ terminal tag of the crRNA and the PAM sequence located upstream of the viral protospacer do not form base pairs. To discriminate between self- and non-self- targets, Cascade screens and specifically binds a PAM sequence, which results in helical destabilization and strand invasion of the matching seed sequence [32,33]. The PAM sequence for type I systems is typically 2–3 bases long and can differ between different subtypes and even organisms. The I-E Cascade complex has a size of 405 kDa and is composed of the five subunits, Cas6e, Cse1, Cse2, Cas7 and Cas5. Cas7 and Cas5 tightly bind and protect the crRNA from degradation [19], whereas Cse1 and Cse2 were shown to be nucleic acid-binding proteins that preferentially interact with the DNA target [34,35].

The targeting complex facilitates base pairing of the crRNA with the complementary DNA strand and additional displacement of the non-complementary strand to produce a so-called R-loop structure [18,36]. Using cryo-electron microscopy, the overall structure of the I-E Cascade complex was resolved and revealed a general outline that is often described as a seahorse-like shape [18,20]. The conservation of the crRNA-binding subunits, Cas7 and Cas5, as well as Cas3, throughout all type I subtypes, suggest structural and functional similarities of Cascade, but further biochemical and structural data are required to compare complexes and the interference reaction in different prokaryotic families in detail. The related I-A Cascade complex shows a conserved Cas7, Cas5 and crRNA assembly platform, which might recruit Cas3 and additional subtype-specific proteins to form the active interference machinery [36,37]. Plasmid-based interference assays were established that could show *in vivo* interference activity for I-A and I-B Cascade modules [38,39].

Type II systems have only been found in bacterial genomes and are characterized by a distinct minimal set of *cas* genes [24,25]. In these systems, the large multifunctional protein, Cas9, is involved in both the maturation of crRNAs and in the subsequent interference reaction [40]. The processing of crRNAs is dependent on a trans-activating crRNA (tracrRNA) encoded in the vicinity of CRISPR loci and containing a 25 nt long stretch that is complementary to the crRNA repeat sequence (Figure 1B) [41]. The comparison of several tracrRNA molecules did not identify any highly conserved sequence or structure elements other than the anti-repeat sequence [42]. Cas9 facilitates the base pairing of tracrRNA and pre-crRNA, which form a RNA duplex that is then targeted by the host endonuclease, RNase III. Cleavage of this duplex by RNase III generates mature crRNAs with 20 nt spacer-derived 5′-tags and 19–22 nt repeat-derived 3′-tags [41,43]. In the interference step, the cleavage of target dsDNA requires not only crRNA and Cas9, but also the presence of tracrRNA. Cas9 cleaves the DNA strand complementary to the crRNA with a McrA/HNH nuclease domain and the non-complementary strand with a RuvC-like (RNase H fold) domain in the presence of Mg^2+^ ions [43]. The interference against the viral DNA requires a conserved 5 nt-long PAM sequence (NGGNG), located immediately downstream of the protospacer [5,44]. The precise DNA cleavage site was identified 3 nt upstream of the PAM for the complementary strand, whereas the non-complementary DNA strand is cleaved at additional sites within three to eight base pairs upstream of the PAM, producing blunt-ended cleavage products [43,45].

The two known type III systems (type III-A and type III-B) are predominantly found in archaeal genomes [24,25], and interestingly, type III-B systems are only found in combination with one or more other CRISPR subtypes. Type III systems encode the CRISPR-specific endoribonuclease, Cas6, and the subtype-specific Cas10 protein that is very likely involved in target interference (Figure 1C). Similar to Cas3 proteins of type I systems, Cas10 encodes a HD nuclease domain that is proposed to have similar function in target degradation [24,25]. The type III-A system of *Staphylococcus epidermidis* contains five Csm proteins and was shown to target DNA [46]. DNA targeting by this system does not require a specific PAM sequence, but sequences complementary to the 8 nt 5′-tag of the crRNA are not targeted by this system [47]. In the type III-B system of *Pyrococcus furiosus*, Cas6 is not an integral part of the interference complex after the crRNA processing, but the 8-nt 5′ repeat tag serves as an anchor for the assembly of a six protein (Cmr1–Cmr6) ribonucleoprotein interference complex. A similar Cmr complex with seven proteins (Cmr1–Cmr7) was identified for *Sulfolobus solfataricus* and shown to endonucleolytically cleave invading RNA at UA dinucleotides [48]. Targeting of RNA was shown to be PAM-independent for both investigated Cmr complexes. The crystal structure of a Cmr2–Cmr3 complex revealed a conserved RNA binding surface of Cmr3, which is reminiscent of the Cas6 RNA interaction surface and illustrates the RNA binding ability of this Cmr subunit [49,50]. Notably, these two interference complexes differ from all other investigated subtypes, as they specifically target RNA and not DNA [11,51]. However, recently, it could be demonstrated *in vivo* that Cmr proteins can target also plasmid DNA in a PAM-independent manner [52].

## 3. CRISPR Systems as Genome Editing Tools

The application of the diverse CRISPR/Cas systems as genetic tools offers great potential. Initially, before the discovery of Cas protein functions, the diversity of CRISPR sequences was mainly utilized in a powerful method to rapidly identify closely related bacterial strains (e.g., *Mycobacterium tuberculosis*). This genotyping method is known as spacer oligonucleotide typing (spoligotyping) [53,54]. The investigation of different Cas protein activities introduced them as diverse components for genetic tool development. One example is the pre-crRNA processing enzyme, Cas6f (previously termed Csy4), which was shown to be useful for predictable gene expression. In these approaches, the Cas6f cleavage site sequence of a CRISPR repeat can be fused to a gene of interest. Processing of this site by Cas6f can then be used to physically separate genetic elements (e.g., untranslated regions, regulatory elements, ribosome binding sites) at the mRNA level. Therefore, transcript impedances (e.g., secondary structures) can be reduced, which results in a more predictable gene expression pattern [55]. Additionally, the high substrate affinity of Cas6f was used to create a specific RNA-binding bait protein. This construct can be used in high-throughput RNA affinity purification protocols to isolate RNA molecules with a 16 nt hairpin sequence derived from the 3′-terminal crRNA repeat tag [56].

The recent analysis of Cas protein interference complexes immediately revealed their great potential for the development of genetic tools that are required to provide specific DNA or RNA targeting. One key player in this development is the large type II protein, Cas9. Qi and colleagues could show that a nuclease inactive mutant of Cas9 in combination with a sequence specific crRNA can be utilized for targeted DNA recognition to interfere with transcriptional elongation, RNA polymerase or transcription factor binding. This gene silencing activity was termed CRISPRi for CRISPR interference in reference to RNAi [57]. Subsequently, other groups reported the utilization of Cas9/crRNA complexes for genome editing in different organisms, e.g., human cell lines, zebrafish, mice, drosophila, yeast and bacteria. Cas9 interference experiments indicated that the fusion product of crRNA and tracrRNA has similar efficiency as the RNase III-processed crRNA:tracrRNA duplex [43]. Therefore, these genome editing approaches use Cas9 together with a specific small guide RNA (sgRNA), which was designed to resemble the fused crRNA/tracrRNA sequence. The resulting ribonucleoproteins are termed RNA guided endonucleases (RGENs) and were shown to target single genes or even multiple genes, allowing efficient and site-specific editing of the target sequence [58–67]. Specificity of the targeting reaction is determined by the sgRNA sequence, which immediately displays the advantage of this method over other established genome editing methods, like zinc-finger nucleases (ZNF) or transcription activator-like effector nucleases (TALEN) [68]. A single Cas9 protein can be retargeted using different sgRNA sequences and without the time-consuming protein engineering steps required for target changes of ZNF and TALEN constructs. Moreover, the utilization of several sgRNAs in one reaction was shown to allow for multiplex editing of five genes [66].

**Figure 2:**
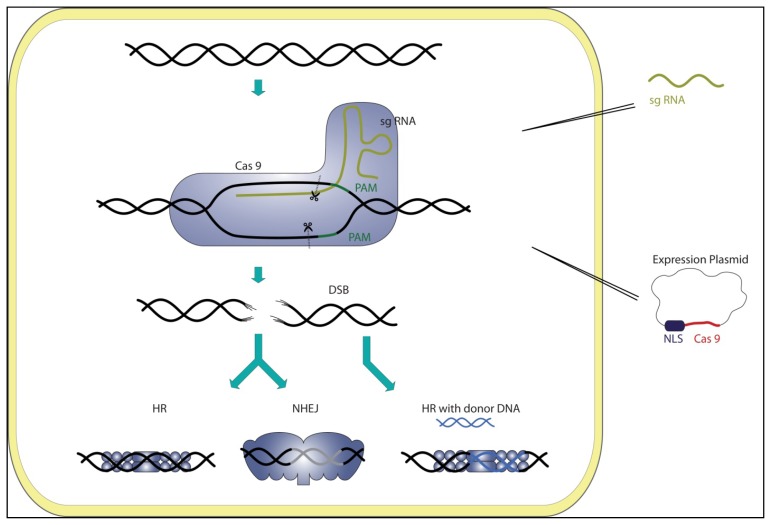
Targeted genome editing using a small guide RNA (sgRNA):Cas9 complex. A plasmid encoding the codon-optimized Cas9 (red) with a nuclear localization signal (NLS) and an sgRNA (yellow), including the desired targeting sequence, are transferred into the target cell. A functional sgRNA:Cas9 interference complex is assembled in the cell. A double strand breaks (DSB) at the targeted DNA sequence upstream of a PAM (green) is introduced by the sgRNA:Cas9 complex, which can be repaired by the host DNA repair mechanisms, homologous recombination (HR) and non-homologous end joining (NHEJ). While HR restores the wild-type sequence by using the template allele, the error-prone NHEJ mechanism leads to insertions and deletions (indels) at the target site (grey). Increased editing efficiency can be achieved by co-transferring a synthetic donor DNA template for a triggered HR (blue).

All reported CRISPR genome editing approaches facilitate sgRNA:Cas9 systems to generate double strand breaks (DSB) of the target sequence, which can be either repaired by homologous recombination (HR) or non-homologous end joining (NHEJ) (Figure 2). In the case of HR, a full repair of the DSB in eukaryotes is facilitated, as the wild-type allele serves as a donor template. In contrast, NHEJ is an error-prone repair mechanism, which leads to the formation of insertions and deletions (indels) that can result in mutations of the particular sequence. The aim of genome editing using RGEN, ZNF or TALEN is to introduce such indels to mutate the gene of interest. However, HR is the preferred DSB repair mechanism *in vivo*, which leads to a lower efficiency of the editing process. To increase the overall yield of edited sequences, a modified donor DNA can be used, which serves as a template during HR (Figure 2).

The basic principles of established RGENs are similar for different cells. In all cases, the sequence of Cas9 was codon optimized for the particular expression system, and a eukaryotic nuclear localization signal (NLS) was fused to the protein. The plasmid encoded Cas9 production in human, mouse and yeast cells was realized by the *in vivo* production of the protein [58–66]. In zebrafish embryo cells, the mRNA of codon-optimized Cas9 fused to an NLS was microinjected before expression [60,62] (Figure 2). A variety of approaches were chosen to import the targeting sgRNA. One of the employed strategies was to transfect plasmids of a codon-optimized Cas9 together with RNase III, pre-crRNA and tracrRNA [58]. Here, it could be shown that host RNAses are sufficient for sgRNA production and that no additional RNase III enzyme is needed. In other experiments, plasmids coding for a single fusion sgRNA were successfully employed [61,64,65]. Additionally, the direct microinjection of a synthesized sgRNA into zebrafish cells or the transfection of *in vitro*-produced sgRNAs in human cells resulted in similar editing efficiencies [59,62]. For the bacterial systems of *E. coli* and *Streptococcus pneumoniae*, HR was exploited with donor template DNA. Here, genomic DNA containing a truncated type II CRISPR system combined with a modified donor DNA template was transformed to yield an edited genome [63,66]. The verification of a successful editing event in mammalian cells was based on the SURVEYOR assay in which a Cel-1 nuclease specifically cleaves mismatches in hybrids of wild-type and mutant PCR products of the targeted sequence region [58,59,62,64,65,69]. Alternatively, successful editing was followed by antibiotic selection in yeast and bacteria cells [61,63].

## 4. Type I and Type III Genome Editing Tools

Currently, only the type II CRISPR/Cas systems are established for genome editing or gene silencing [58–66]. What are the barriers that prevent the development of type I or type III editing tools? One difference between the three major CRISPR/Cas types is that the interference reaction of both, type I and III systems, relies on multi-protein complexes. This complicates the transfer of these systems to other organisms, and protein-engineering of a single Cas9 protein is more straightforward than optimization of Cascade or Cmr/Csm complexes would be [11,18]. However, the fact that type III systems do not need PAM sequences for interference should be advantageous for more versatile editing events [32]. One restriction of RGEN genome editing tools is the PAM sequence, which is needed for interference. Therefore, this methodology enables the editing of sequences that occur on average every 8 bp, which leads to approximately 40% of the exons in the human genome being applicable for targeting [58,65,70]. Without the restricting PAM sequences for the interference in type III systems, genome editing could, in principle, be accomplished for any given target sequence. However, it should be noted that less sequence restriction needs to be in balance with specificity, as targeting of additional unwanted sequences has to be avoided. Similar to established bacterial editing systems, where truncated type II CRISPR/Cas systems were transformed, an approach using minimal type I (e.g., type I-F) or type III interference cassettes in combination with an engineered CRISPR cluster is conceivable. Nevertheless, the interference of a type I and, especially, type III CRISPR/Cas system and their minimal Cas protein assemblies are still not fully understood. Future research will address their potential role as genome editing tools.

## 5. Genome Editing Tools: RGEN, TALEN, ZFN

The advantages of RGENs over other genome editing tools rest upon the fast, simple and economical design of the targeting crRNAs and the usage of multiple small RNAs for multiplex engineering of several genes [58,62,63,65,66,70]. The ZiFiT Targeter program was designed as a tool to easily find potential sgRNA:Cas9 targeting sites in a given genome [62]. RGEN editing works at a similar efficiency compared to ZFN or TALEN, and so far, no off-site targeting was reported [59]. A further advantage of RGENs is that the CRISPR interference results in a selection for positive clones, as wild-type sequences are constantly targeted until the mutated donor DNA is integrated by HR and inhibits the interference process [61]. Other features of the RGEN approach reveal their restrictions and disadvantages in genome editing. One problem of RGENs is their dependence on a small PAM sequence, which restricts the choice of potential target sequences. Furthermore, the length of the crRNA and its seed sequence limit the range of target sequences and, thus, an optimal design of the sgRNA is required for efficient editing [59,62,64]. ZFN and TALEN systems require protein-engineering steps, but both systems are highly tunable by exchanging the particular domains for sequence specific recognition of the target. Furthermore, libraries of different proteins already exist [71], which reduces the time and costs for the generation of new functional proteins [59,72]. The modular structure of the two systems (TALEN and ZNF) yields a highly variable tool in which the effector domain can be fused to a variety of different proteins (e.g., transposases, nucleases, transcriptional regulators) [73–75]. ZFN- and TALEN-based gene therapy approaches are tested for clinical use. Further improvements, e.g., considering recent advances in re-organizing chromosomes using RGENs, will extend the number of possible applications in gene therapy [72,76].

## 6. Outlook

The potential of CRISPR/Cas systems to function in genetic tools has been discussed since the identification of the system as a prokaryotic immune system. Six years after its discovery, the first steps have been made, as sgRNA:Cas9 complexes are used for efficient genome editing [58–66] and the establishment of gene silencing [57]. Development of RGEN-based genome editing systems for further model organisms (e.g., plants, insects, Archaea) could simplify their future genetic manipulation. It is required to further increase the efficiency and elucidate the occurrence of off-site targeting of RGENs to turn this system into a potential tool for disease treatment, as it has been, e.g., achieved with ZFN approaches for the treatment of HIV [72]. The establishment of RGEN genome editing tools is only one aspect, in which CRISPR/Cas immunity shows its potential for application in genetic and biotechnological systems. Other approaches include the development of phage-resistant bacterial strains used in industrial processes or the identification of the interaction of pathogenic bacteria in a clinical setting with either phages or conjugative plasmid that can transfer antibiotic resistance. Future research on basic mechanistic details of the different CRISPR/Cas systems will reveal a more complete picture of the extensive applicability of these immune complexes.
